# Application of Bayesian Networks and Information Theory to Estimate the Occurrence of Mid-Air Collisions Based on Accident Precursors

**DOI:** 10.3390/e20120969

**Published:** 2018-12-14

**Authors:** Rosa María Arnaldo Valdés, Schon Z.Y. Liang Cheng, Victor Fernando Gómez Comendador, Francisco Javier Sáez Nieto

**Affiliations:** 1 Department of Sistemas Aeroespaciales, Transporte Aéreo y Aeropuertos, School of Aerospace Engineering, Universidad Politécnica de Madrid (UPM), Plaza Cardenal Cisneros n3, 28040 Madrid, Spain; 2 Aeronautic, Space & Defence Division, ALTRAN Innovation S.L., Calle Campezo 128022 Madrid, Spain; 3 Centre for Aeronautics, School of Aerospace, Transport and Manufacturing, Cranfield University, Cranfield MK43 OAL, UK

**Keywords:** aviation safety, loss of separation, Bayesian network approach, information theory, entropy

## Abstract

This paper combines Bayesian networks (BN) and information theory to model the likelihood of severe loss of separation (LOS) near accidents, which are considered mid-air collision (MAC) precursors. BN is used to analyze LOS contributing factors and the multi-dependent relationship of causal factors, while Information Theory is used to identify the LOS precursors that provide the most information. The combination of the two techniques allows us to use data on LOS causes and precursors to define warning scenarios that could forecast a major LOS with severity A or a near accident, and consequently the likelihood of a MAC. The methodology is illustrated with a case study that encompasses the analysis of LOS that have taken place within the Spanish airspace during a period of four years.

## 1. Introduction

Although, during the last decade, mid-air collisions (MAC) between large commercial aircraft have been rare events, maintaining safe separation between aircraft is one of the key aviation safety challenges as the new generation of air traffic management (ATM) systems (SESAR and NextGen) develops. Although traditionally included on the “Significant 7” list of safety risks derived from analysis of worldwide fatal accidents and high-risk occurrences, in 2017 EASA declared airborne collisions the top safety priority from an ATM perspective [1]. 

However, MAC are rare, so relevant data are scarce. Because of the low-frequency, high-consequence nature, MAC are not well represented by conventional statistical models. In the absence of sufficient accident direct data, precursor-based probabilistic risk analysis methods are considered a promising and efficient tool for this purpose [2]. The widely-accepted definition of an accident precursor is an event with no catastrophic or severe consequences but that could have developed into an accident if additional safety barriers had failed [3,4,5,6,7]. 

The significance of precursors in the analysis of major accidents has been explored in several safety-critical industrial sectors, such as space shuttle explosions [6], nuclear power accidents [8,9]; gas and oil accidents [10]; transport accidents [11,12]; etc. 

Since major accidents are frequently preceded by a number of accident precursors, there is a huge opportunity to reduce the risk of MAC by improving insight into MAC’s main precursors, known as LOS or ’loss of separation’ events, as LOS events occur more often in airspace without necessarily having adverse or catastrophic consequences.

A LOS between in-flight aircraft happens when the safety separation minima prescribed in a controlled airspace by ATS (air traffic services) authorities, according to ICAO (International Civil Aviation Organization) standards, are not observed. Different degrees of severity are established depending on the seriousness of the consequences of the LOS. The severity of a LOS is defined by the risk of collision (risk of ending up in a MAC), according to the minimum achieved separation between the involved aircraft and their rate of closure. Eurocontrol [13] has established five levels of severity that range from the most severe, A, “Serious incident”—i.e., a high risk of collision—to the less severe E, “No safety effect”; with intermediate levels being B, “Major incident”; C, “Significant incident”; and D, “Not determined”.

Recent increases in reported losses of the required minimum in-flight separation between aircraft produced sufficient alarm to persuade all interested parties to urge occurrence reporting and share the outcomes of the resultant studies in order to improve mitigations. According to the Airborne Conflict Safety Forum, there are approximately 150 losses of separation per million flights in European-controlled airspace [14]. Considering that on average each flight receives 15 air traffic controller instructions while flying en route, this signifies one loss of separation per 100,000 air traffic controller instructions. 

Although the numbers for LOS are small compared to traffic volume, they are still regarded as critical safety indicators. Because of the severity of its potential consequences, LOS is considered the main proxy and a precursor to a potential MAC, and consequently the analysis of contributing factors and the multi-dependent relationship between causal factors of LOS incidents is encouraged as an effective way to mitigate LOS instances and prevent MAC. 

EASA has recently produced an industry best practice document identifying relevant LOS precursors (contributing factors and the multi-dependent relationship between causal factors) to be monitored through FDM (flight data monitoring) programs [15]. However, this work only focuses on those precursors that can be monitored from the data recorded on board. The investigation of LOS occurrences and their precursors from an ATC (air traffic control) point of view is not so ingrained, in part due to the inherent complexity of such incidents and in part due to the scarcity of information available for their detailed analysis. To compensate for this partial approach, this research is constructed from the official reports of investigations of LOS occurrences produced by the official States Incident Investigation Authorities.

This paper relays on the combined used of Bayesian networks (BN) and information theory to model the probability of severe LOS near accident occurrences. In a first step, the BN models LOS contributing factors and allows the analysis of the multi-dependent relationship between them. The BN model is widely used for risk analysis [16,17] and decision-making [18,19] in complex systems as well as the ATM system. The uncertainty presented in LOS scenarios makes the application of the BN model the preferred candidate for this study. In a second step, information theory is used to identify the LOS precursors that provide the most information. The combination of techniques permits the use of LOS causes and precursors to delineate perceptive warning scenarios that could forecast a major LOS or near accident, and therefore anticipate a MAC accident. 

The methodology is applied, as a case study, for the analysis of the LOS during a period of four years in Spanish airspace.

The present work is aimed at exploring BN and Information Theory methodologies for precursor-based risk analysis of a category of major accidents in aviation known as MAC. The proposed method combines principles from quantitative risk analysis, Bayesian modeling, and information theory, to infer the likelihood of catastrophic accidents based upon precursor data.

## 2. Materials and Methods

**Figure 1:**
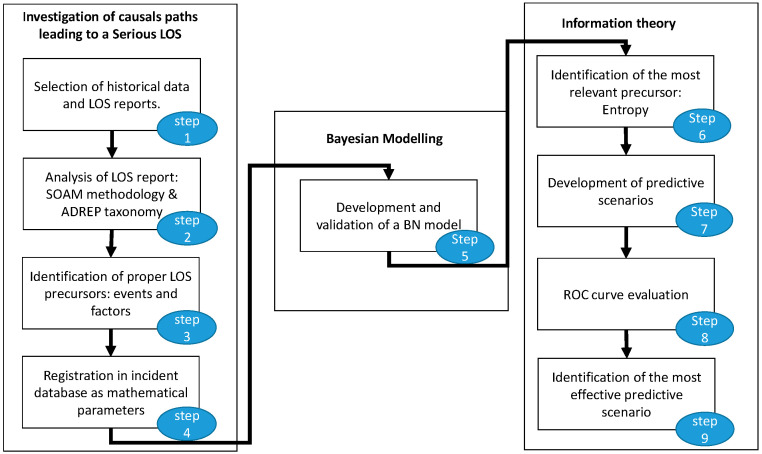
Steps in the methodology.

The proposed methodology follows the main phases and steps indicated in Figure 1.

The starting point is the investigation of causal paths leading to a serious LOS. The objective of this first phase is to identify precursors leading to a LOS serious incident from the analysis of the occurrence notification and investigation reports. During this phase, data collected from serious incident reports are filtered into events and factors following a determined procedure of analysis; both are interpreted as precursors to accidents that might occur in the future. Standardized taxonomies and analysis methodologies are applied in this process. 

In a second phase, the correlation between events and factors is used as the basis for the development and validation of a BN model. The BN model provides a quantitative cause‒effect map that reproduces serious LOS scenarios. This model contains the known relationship that was detected by previous researchers and the new relationships that are established as the target of this model, as well as the estimated likelihood based on the number of reports investigated.

In a third phase, information theory principles and the concept of entropy are used to identify the precursor (events and factors) most correlated to when a serious LOS incident occurs. In the last phase, this information is used to define predictive scenarios and the most effective predictive scenario is evaluated using a ROC (receiver operating characteristic) curve.

The preceding steps are described in more detail in the following sections.

### 2.1. Investigation of Causals Paths Leading to a Serious LOS

The first step (Step 1) in this process accounts for the selection of historical data and LOS reports. The reporting and evaluating of occurrences are of prime importance in safety analysis, as well as investigations after the fact. They provide necessary information for identifying safety-related trends and foreseeing emergency safety risks [20]. 

According to European Regulation (EU) no. 376/2014 [21], pilots, air traffic controllers, airport managers, aviation maintenance technicians, and aircraft ground handlers are mandated to report occurrences to the competent authorities. According to ICAO Annex 11 [22], all air traffic LOS should be investigated by the state where they took place. In Spain, air traffic incidents are reported to a State Investigation Office where the incidents data is analyzed and compiled for publication in reports [23]. This research builds upon the data collected from LOS investigation reports that contain information related to the severity of the LOS occurrence, contextual and factual data, and results of the assessment/investigation as required by aviation regulations. The incident reports include not only LOS incident scenario data, but the testimonies of involved agents and recommendations given by the investigating office. In this study, a period of four years of occurrences and reports are being considered.

The second step (Step 2) accounts for the analysis of those reports and the identification of proper LOS precursors. Standardized analysis methodologies and taxonomies are applied in this process.

In the analysis process, the SOAM approach [24] is employed for incident report analysis, and factual data are processed with criteria defined in EAM 2/GUI 8 [25] to identify adverse events and influential factors, which are extracted and encoded by applying ADREP taxonomy [26]. 

The adverse events have a direct correspondence with ADREP taxonomy [27] as events, while the influential causes extracted from reports correspond to DFs (descriptive factors) and EFs (explanatory factors). In a cause‒effect relationship, events are interpreted as effects or stages that set in motion the incident. Both DFs and EFs are causes of failures. The three components: “events, DFs and EFs” are identified as “precursors” (Step 3).

As a result of this process, incident reports are thus transformed from texts to simple cases formed by events and factors, or precursors, which could be registered in an incident database as mathematical parameters (Step 4).

Within this database events and their associated factors from all analyzed incidents can be grouped together. Taking advantage of this result, a map of the correlation between both groups is depicted that simplifies the causal model construction. Additionally, the same model attempts to achieve a predictive feature to determine adverse events and influential causes in future ATM incidents.

The whole procedure is focused on providing a chronological vision related to incident scenarios that can be separated by events and factors. Hence, the traceability between report and analysis is preserved.

### 2.2. Development and Validation of a BN Model

Our endeavor is that our model would have a predictive feature to determine the adverse events and associated causes in future ATM incidents. In a similar situation of complex system modeling, researchers Wilson & Huzurbazar [28] and Khakzad [29] suggested that conventional safety models, like the FT (fault tree) model, do not offer enough capacity to capture the specific features of this kind of system, so a BN model is proposed (Step 5).

A BN is a probabilistic graphic approach used to provide a mathematical method related to the detection of uncertain variables. A BN model consists of a DAG (directed acyclic graph), which reflects the relationship between a set of stochastic variables, also identified as nodes, and arcs, which represent probabilistic or functional influence linking two nodes [30]. The strength of the connections between both nodes is denoted by the CPT (conditional probabilistic table) [31].

The BN also represents a joint probabilistic distribution *P*(*U*) of variables *U* = {*A*1, *A*2, *A*3, …, *An*}. Such distribution could be continued or discrete, based on the conditional independency and chain rule [32] included in the network as

(1) $$P\left(U\right)=\prod_{i=1}^{n}P\left(\left(A_{i}|\mathrm{Pa}\left(A_{i}\right)\right)\right),$$

where Pa(*Ai*) is the parent set of *Ai* and *P*(*U*) is the joint probabilistic distribution in BN.

For LOS precursor analysis, the BN applies the Bayes theorem to update the prior occurrence probability of events or factors, depending on the levels in consideration [29], providing new inputs called evidence *E* to yield the posterior consequence probability by applying the next equation

(2) $$P\left(U|E\right)=\frac{P\left(U,E\right)}{P\left(E\right)}=\frac{P\left(U,E\right)}{\sum_{U}P\left(U,E\right)}.$$

Equation (2) demonstrates either probability prediction or probability updating. In predictive analysis, conditional probabilities of *P*(event|factor) are calculated, specifying the probability of a particular event when the occurrence of a specific factor is known. In updating the analysis, the *P*(factor|event) is evaluated, showing the occurrence of a particular factor when the occurrence of a specific event is known [33]. In fact, the values of *P*(factor|event) are calculated directly and collected in CPT; on the contrary, the values of *P*(event|factor) can be estimated with GeNIe software [34]. Additionally, all events and factors are defined either as present state or absent states in this BN model.

In a CPT, each event can be associate with one or more factors. This evidence infers that behind a LOS there are one or more supporting causes.

### 2.3. Identification of the Most Relevant Precursors Through Information Theory Principles

MAC accidents and LOS have common causes or contributing factors in the form of initiating events and factors. The occurrence of a LOS would imply changes in the probabilities of the common causes (events and factors) that in the end could affect the probability of a catastrophic accident. That is, the occurrence of a LOS and its causes would include information about the occurrence of the final accident that can be quantified using the concept of mutual information. Among the causes of a LOS, those with the highest mutual information with high severity LOS are more informative, i.e., if this presents itself, it reduces the uncertainty about the potential occurrences of major accident (Step 6).

If we consider a LOS as a random variable with probability mass function of *P*(*z*), then the amount of uncertainty associated with the values of z can be measured by the entropy *H*(*z*)

(3) $$H\left(Z\right)=-\sum_{z\in Z}P\left(z\right)logP\left(z\right).$$

The conditional entropy of *Z* given the cause *Y* (any combination of event and factor) is also a random variable defined as

(4) $$H\left(Z|Y\right)=-\sum_{z,y}P\left(z\right)log\frac{P\left(z,y\right)}{P\left(y\right)}.$$

The mutual information of Z and *Y*, *I*(*Z*,*Y*) can be defined in the uncertainty of *Z* given the observation of *Y*

(5) $$I\left(Z,Y\right)=H\left(Z\right)-H\left(Z|Y\right)=\sum_{z,y}P\left(z,y\right)log\frac{P\left(z,y\right)}{P\left(z\right)P\left(y\right)}=\sum_{z,y}P\left(y\right)P\left(z|y\right)log\frac{P\left(z|y\right)}{P\left(z\right)}.$$

The calculation of conditional probabilities is straightforward from the corresponding BN, which allows a quick and easy update of the mutual information when new data become available.

### 2.4. Development and Evaluation of Predictive Scenarios

The identification of the most informative contributing factors (events and factors) can be used to establish the probability of a major accident. The most informative contributing factors will be used as a binary classifier.

Considering the most informative contributing factors, different predictive scenarios can be developed (Step 7) and their performances are examined by a ROC curve (Step 8), a graphical tool to determine the performance of a model—in particular a binary classifier, based on discrimination threshold. By performing this analysis, the most effective predictive scenario is identified (Step 9).

**Figure 2:**
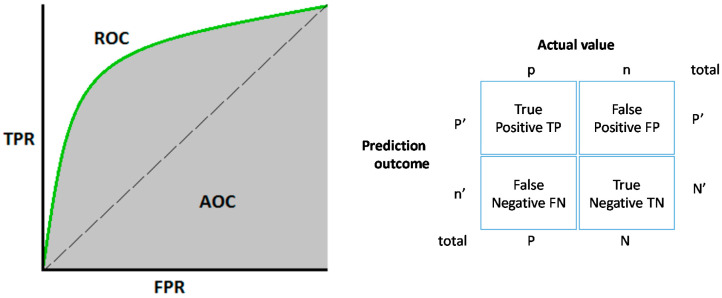
ROC Curve for a binary classifier.

A ROC curve, as indicated in Figure 2, presents the true positive rate (TPR) versus the false positive rate (FPR). Given a specific threshold, TPR is the ratio of true positives out of total actual positives as indicated in Equation (6). FPR is the ratio of false positives out of total actual negative as indicted by Equation (7).

(6) $$\mathrm{TRP}=\frac{\mathrm{TP}}{\mathrm{TP}+\mathrm{FN}}$$

(7) $$\mathrm{FPR}=\frac{\mathrm{FP}}{\mathrm{FP}+\mathrm{TN}}$$

TP is true positives, FP is false positives, FN is false negatives, and TN is true negatives. 

The ROC curve is defined by FPR on the horizontal axis and TPR on the vertical axis. The diagonal line, known as the line of no discrimination, divides the space into three areas. The space above the no discrimination line represents good predictions. The TPR is also known as ’sensitivity’ or ’recall’. 

The points below the no discrimination line represent poor predictions. The points along the line of no discrimination represent a random result. Finally, the accuracy of the classifier can be defined as

(8) $$\mathrm{ACC}=\frac{\mathrm{TP}+\mathrm{TN}}{\mathrm{FTP}+\mathrm{FP}+\mathrm{TN}+\mathrm{FN}}.$$

## 3. Case study: Assessment Four Years of LOS in Spanish Airspace

To illustrate the application of this methodology, a period of four years of LOS occurrences and reports is considered in this study. As summarized in Figure 1, the application of the proposed methodology implies three mayor phases with nine steps in total. For the sake of clarity, this section is structured in three main subsections, which address the steps in each phase:

- Phase 1: Investigation of causal paths leading to serious LOS
- Phase 2: Bayesian modeling
- Phase 3: Information theory

### 3.1. Case Study Phase 1: Investigation of Causal Paths Leading to Serious LOS (Steps 1 to 4)

**Figure 3:**
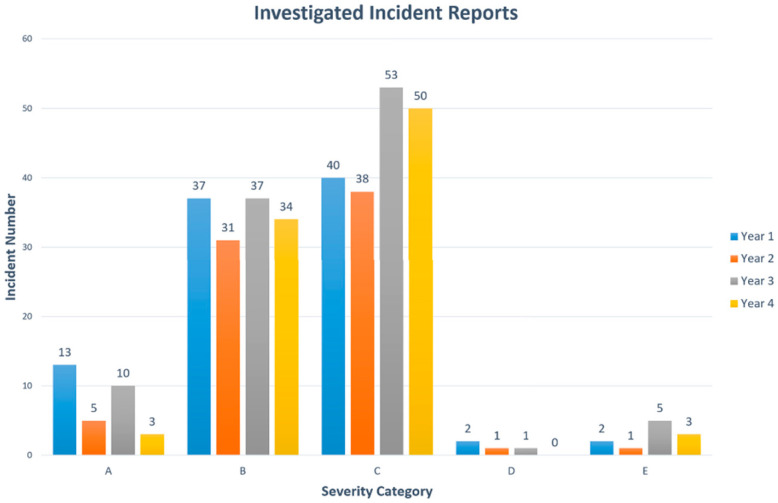
Spanish investigated incidents during four consecutive years.

The first step in this process is the selection of historical data and LOS reports (Step 1). In Spain, air traffic incidents are reported to the authorities and incident data are analyzed and compiled for publication [23]. These reports consist of all ATM incidents scenarios, gathering the testimonies of implicated individuals, the investigation’s conclusions, and recommendations for affected entities. Figure 3 presents the number of categories and occurrences of all reported incidents during four consecutive years {U}. In our research, the data collected with ATM reported incidents are composed of

$$\left\{U\right\}=\left\{U_{anal.}\right\}+\left\{U_{ass.}\right\}$$

where $\left\{U_{anal.}\right\}$ is data collected from the first three years as an analysis dataset for BN modeling and analysis, and $\left\{U_{ass.}\right\}$ is data collected from the last year as an assessment dataset to study.

As a result of the analysis (Step 2) of those reports, causal paths leading to a Serious LOS are outlined and precursors leading to a loss of separation serious incident are identified and filtered into events and factors (Step 3), and registered in an incident database as mathematical parameters (Step 4).

### 3.2. Case Study Phase 2: Bayesian Modeling (Step 5)

The BN model proposed in this work (both the structure and the numerical probabilities) is based on a combination of expert knowledge and objective frequency data. The proposed BN model was constructed using GeNie software. There are several stages in the BN model to assess the risk of MAC precursors.

**Stage 1**: extract the key factors causing LOS and determine the BN nodes. Determination of nodes is the foundation and key for determining the structure of the BN. The nodes in the network correspond on one side to the precursors of a LOS, and on the other side to the type of LOS resulting at each incident. There are three categories of nodes taken into account.

- Adverse events, that is, effects or stages that set in motion the LOS incident.
- Influential causes extracted from reports. Influential factors can be either DFs (descriptive factors) or EFs (explanatory factors). Both DFs and EFs are causes of failures. The three components events, DFs and EFs are identified as precursors.
- The type of LOS resulting at each occurrence. LOS are classified according to its severity.

During the BN model construction for this case study, not all factors identified in incident reports were considered valid for data processing. Due to incidents not being as strictly investigated as accidents, the EFs have rarely been collected, thus the reliability of our case study would be damaged. Therefore, only DFs are considered for this BN modeling. Events and factors in the BN model have been divided into five groups:

- Group 1 of events, parent nodes, related to A/C systems or flight crew’s operations.
- Group 2 of events, parent nodes, related to ATM systems or operations.
- Group 3 of factors, children nodes, related to A/C systems or flight crew’s operations.
- Group 4 of factors, children nodes, related to ATM systems or operations.
- Group 5 of factors, children nodes, related to the interaction of operations between flight crew and ATM.

The conditioned independence of each node and the dependency relationship between parent and child nodes are assumed. The explicit hypothesis is P (*xi*|*x*1 ⋯ *xi* − 1) = P (*xi*|parents (*Xi*)), that is, a DF is conditionally independent of the other DFs given an event (parent node). In addition, because of the characteristic of taxonomy and each factor or event is a taxon, then these factors or events are independent of each other. Finally, regarding the default options, all the nodes in the network are defined as chance-general. 

**Stage 2**: Determine the BN structure. The structure of BN consisted of a causality chain, derived from logic analysis and expert knowledge. 

The analysis in the first phase of the case study (Steps 1 to 4) is used to build the BN structure. During this phase the identified events and factors are registered in an incident database as mathematical parameters, and the correlation between events and factors is used as the basis for the development and validation of a BN model (step 4).

**Stage 3**: Instantiate the BN with probabilities. Prior probabilities are assigned to the root nodes and, next, conditional probabilities are assigned to other nodes. It is tough to gather much information about incidents and causal factors in actual civil aviation operation, and sometimes experts have trouble providing much information. To solve the problem, the BN was analyzed for simplifying the assignment of conditional probabilities.

Prior probabilities are assumed to follow a multinomial distribution, with the parameter vector $\theta_{1},\theta_{2}$,…, $\theta_{n}$ where *n* is the number of states of variable *x* and $\theta_{k}=P\left(x=x_{k}|p\right)$, for 1≤k≤n*x*; where θ posses the Dirichlet distribution $\theta ∼D[\propto_{1},\;\propto_{2},\ldots ,\;\propto_{n}]$, and $\propto_{i}>0;i=1,\ldots ,n$ and $\sum_{i=1}^{n}\theta_{i}=1$, $\propto_{i}$ representing counts of past cases that are stored as a summary of experience in the database produced in Step 4.

For belief updating in the Bayesian network, as a default option, we have used the most popular inference algorithm offered by GeNIe, the “clustering algorithm”. A clustering algorithm is the fastest exact algorithm for belief updating in Bayesian networks and is sufficient if the network is not very large or complex. It produces marginal probability distributions over all network nodes and works in two phases: (1) compilation of a directed graph into a junction tree, and (2) probability updating in the junction tree. 

**Stage 4**: Learn BN structure. There are two ways of learning in BN structure. One involves deciding the BN structure by data reasoning. The other consists of verifying the structure of BN and remove weak connections between nodes by massive data sets. In our model, the initial BN structure has been decided based on expert knowledge and the study of Phase 1.

**Figure 4:**
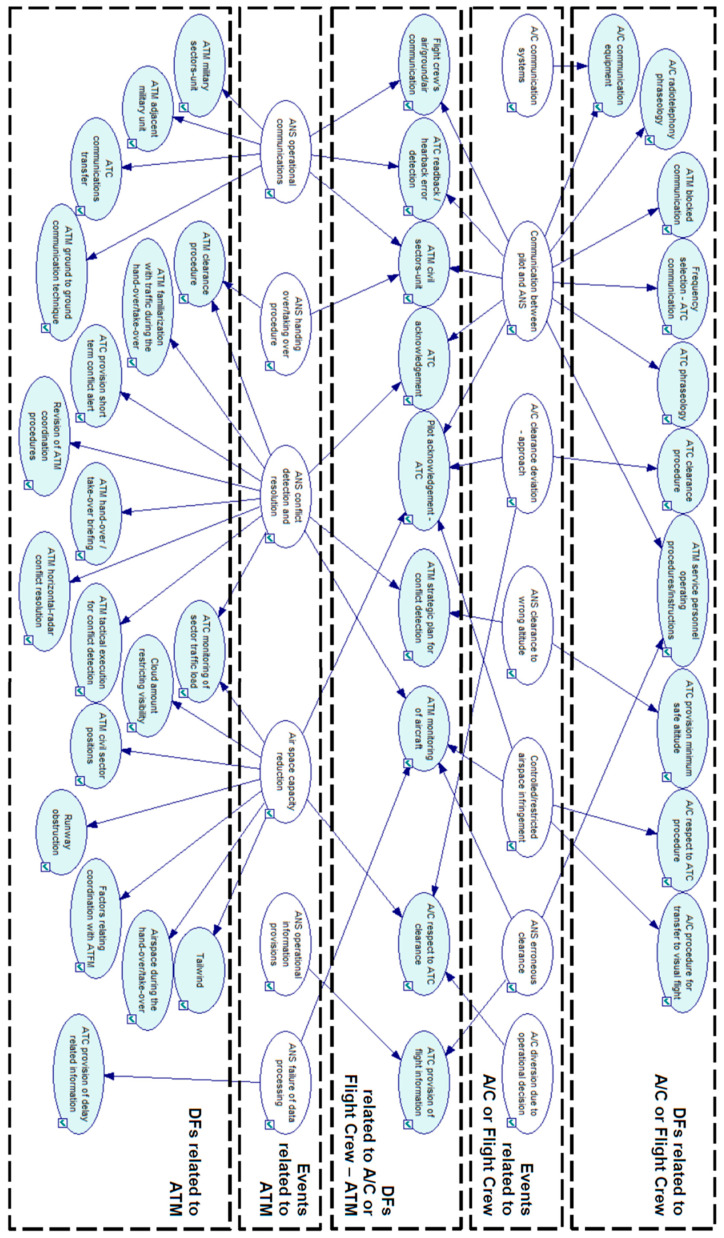
BN model for loss of separation serious incident in civil/commercial aviation.

**Figure 5:**
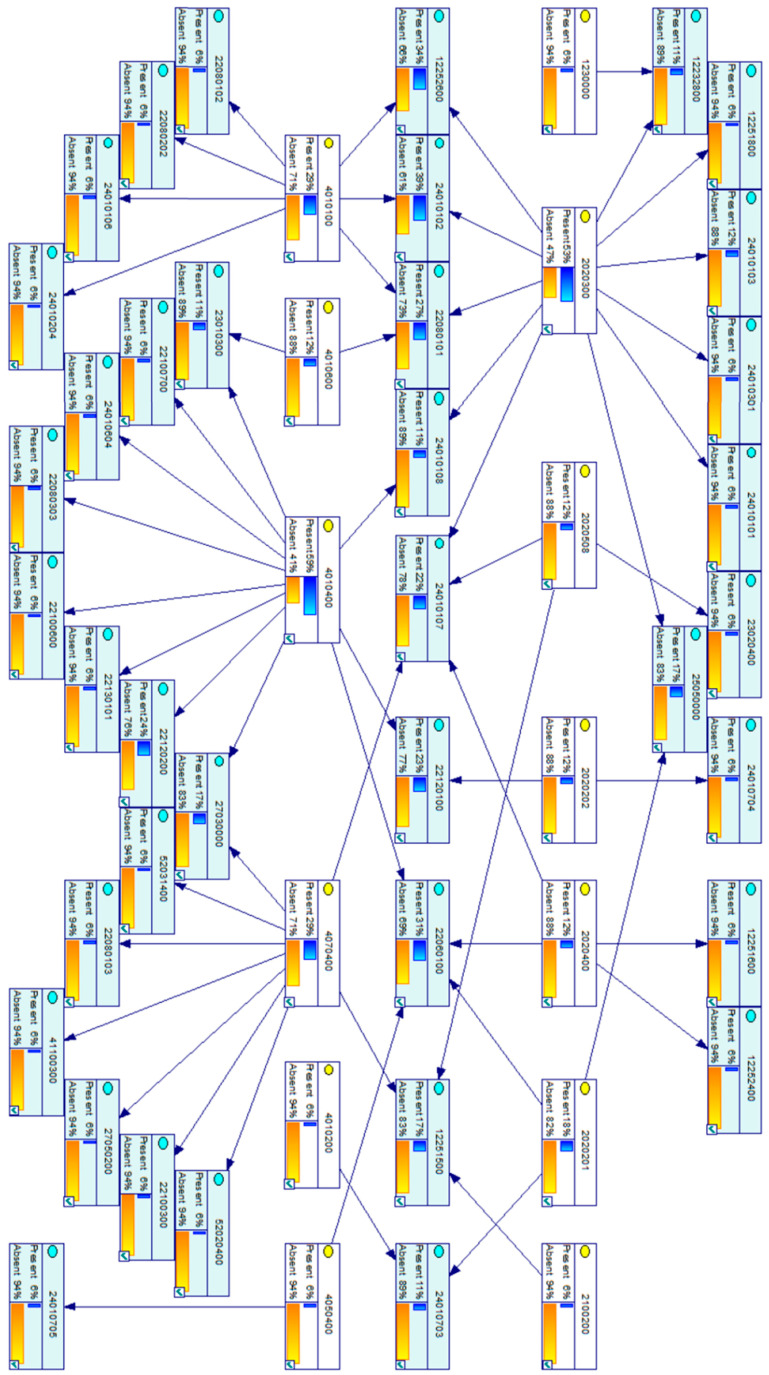
GeNIe output of events and DFs during first three years.

The CPT of the correlation between events and DFs into a loss of separation scenario is summarized in Table 1 with the analysis dataset $\left\{U_{anal.}\right\}$; its derived DAG is represented in Figure 4 as a correlation map. The proposed BN model is illustrated in Figure 5. The assessment dataset $\left\{U_{ass.}\right\}$ is collected in Table 2 and used for BN model application analysis.

**Stage 5**: Learn BN parameters. The Bayes method uses prior density and posterior density to learn and assess parameters. BN also uses the above process to learn parameters after collecting and accumulating relevant data. In the practical application, the parameter learning of BN also uses conjugates prior to simplify parameter learning.

**Stage 6**: Validation. The BN is validated using the validation functionality of the GeNIe software [35]. Three alternatives are available: a) test only, b) K-fold cross validation, and c) leave one out. The simplest evaluation is test only, which amounts to testing the model on the data file and is suitable for situations when the model has been developed based on expert knowledge. If we want to both learn and evaluate the model on the same dataset, the most adequate evaluation method is cross-validation. GeNIe implements K-fold cross-validation, considered the most powerful cross-validation method. It divides the dataset into K parts of equal size, trains the network on K-1 parts, and tests it on the last, Kth part. The process is repeated K times, with a different part of the data being selected for testing. K-fold cross-validation was selected with the number of folds K = 10. The model evaluation technique implemented in GeNIe keeps the model structure fixed and relearns the model parameters during each of the folds. One data file of 1000 records is generated by applying this software and used to run the BN model validation. The validation accuracy of all 51 nodes is 0.956 and for individual node it is calculated with GeNIe.

GeNIe also allows for using the leave one out (LOO) method, which is an extreme case of K-fold cross-validation in which K is equal to the number of records (N) in the dataset. In LOO, the network is trained on *n* − 1 records and tested on the remaining one record. The process is repeated *n* times. 

**Figure 6:**
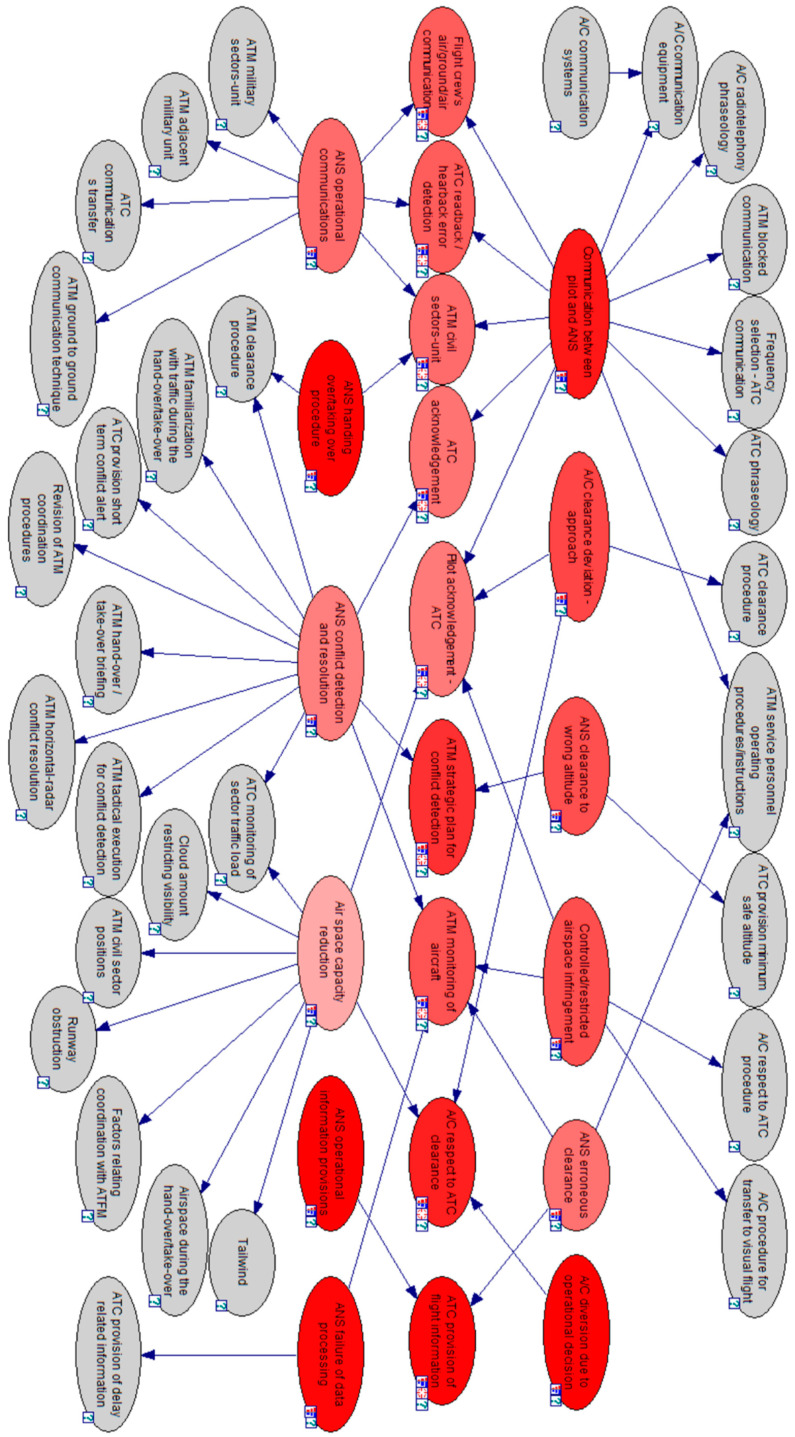
Results of the sensitivity analysis.

**Stage 7**: Sensitivity analysis. A simple sensitivity analysis is done to identify highly sensitive parameters that affect the reasoning results significantly. The analysis is done with the GeNIe by the default algorithm, proposed by Kjaerulff and van der Gaag. Figure 6 presents the results of the analysis, showing in dark red the most sensitive parameters of the network.

**Stage 8**: Infer BN. The inferential analysis consists of two parts:

1. Forward conditional probability approach, introducing events of assessment dataset $\left\{U_{ass.}|Event_{i}\right\}$ in the BN model to determine DFs and compare this result with real data $\left\{U_{ass.}|DF_{i}\right\}$;
2. Backward conditional probability approach, introducing DFs $\left\{U_{ass.}|DF_{i}\right\}$ of assessment dataset in the BN model to determine events and compare the results with real data $\left\{U_{ass.}|Event_{i}\right\}$.

### 3.3. Case Study Phase 2: Information Theory (Steps 6 to 9)

**Figure 7:**
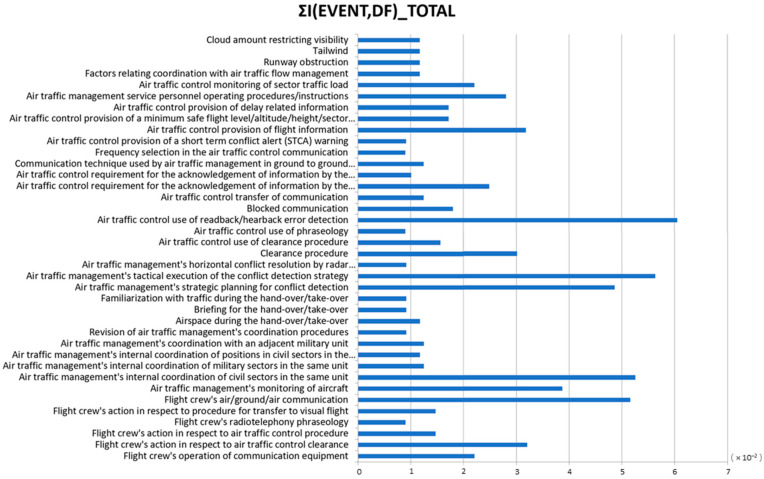
Mutual information for each descriptive factor in all LOS events.

Once the BN has been validated, we could apply the entropy principle to identify the events and descriptive factors with the biggest contribution to a LOS. Using Equation (7), the mutual information of the LOS severe incidents and events/descriptive factors are calculated and presented in Figure 7 (Step 6). As can be seen, compared to other combinations of events-DF, the occurrence of the following DFs conveys the most information following the occurrence of a high-severity LOS:

- DF 24010102: Air traffic control use of readback/hearback error detection
- DF 22120200: Air traffic management’s tactical execution of the conflict detection strategy
- DF 22080101: Air traffic management’s internal coordination of civil sectors in the same unit
- DF 12252600: Flight crew’s air/ground/air communication
- DF 22120100: Air traffic management’s strategic planning for conflict detection
- DF 22060100: Air traffic management’s monitoring of aircraft

Considering the previous DFs as the most informative precursors and using them as the predictive classifier, different predictive scenarios are developed (Step 7); their performances can be examined using the ROC curve and in particular the TRP value. 

A total of 10 scenarios have been defined. The first six correspond to each of the precursors independently. The seventh scenario corresponds to the combination of two precursors; the eighth and ninth scenarios correspond to the combination of three precursors and, finally, the last scenario corresponds to the combination of the six precursors previously identified. 

The way to interpret the scenarios is as follows. In Scenario 1 the DFs “24010102: Air traffic control use of readback/hearback error detection” is used to predict the occurrence of a LOS. The values of TPR and FPR for this predictive classifier are 0.42, and the Accuracy (ACC) of the classifier is 0.58. TPR and PFR values are calculated according to Equations (6) and (7), where:

- TP is the number of times that both the classifier “24010102: Air traffic control use of readback/hearback error detection” and a severe LOS took place.
- FN is the number of times that the classifier “24010102: Air traffic control use of readback/hearback error detection” did not take place although a severe LOS occurred.
- FP is the number of times that the classifier “24010102: Air traffic control use of readback/hearback error detection” took place but no severe LOS occurred.
- TN is the number of times that neither the classifier “24010102: Air traffic control use of readback/hearback error detection” took place nor the severe LOS occurred.

The interpretation of Scenarios 2 to 6 is equivalent. In Scenario 7, the classifier corresponds to the simultaneous presence of two descriptive factors (24010102+22060100: Air traffic control use of readback/hearback error detection & Air traffic managements’ monitoring of aircraft). The values of TPR, FPR, and ACC for the classifier in this scenario are 0.74, 0.16, and 0.84, respectively. The interpretation of Scenarios 8, 9, and 10 is equivalent, although the classifier is based on the occurrence of three DFs and six DFs depending on the case. 

**Figure 8:**
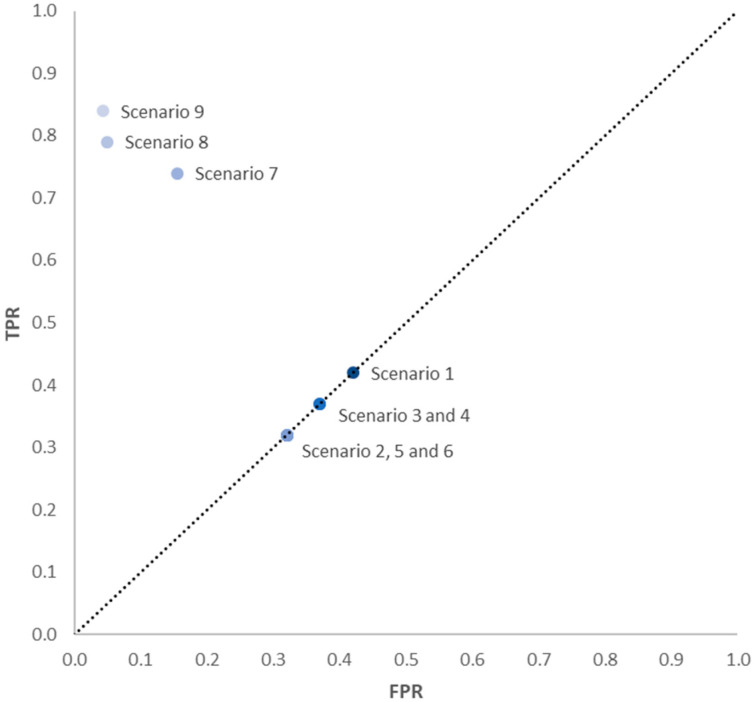
ROC curve to show the efficacy of precursor data in the prediction of LOS.

Table 3 summarizes the values of TPR, FPR, and ACC for each of the defined scenarios. The value of TPR reflects the integrity of the classifier, i.e., the conditional probability of correctly classifying/predicting the occurrence of the losses of separation. The prediction accuracies of aforementioned scenarios are depicted by the value of ACC. Additionally, Figure 8 shows the ROC curve analysis for all defined scenarios. It can be observed how Scenario 9 provides high values of TPR and ACC with just three precursors or descriptive factors. 

These results lead to great operational usefulness. Based on these results, it is possible to implement a monitoring program of the air traffic controller’s activity during normal operations. By monitoring the occurrence of identified DFs, it will be possible to anticipate or predict the occurrence of a high-severity LOS. This program will be extremely cost-effective; instead of complicated and wide supervisory programs, it will only require the monitoring of a few precursors during the air traffic controller activity—those that have the highest mutual information with the LOS.

## 4. Conclusions

In this work, the authors have developed a method that combines principles from quantitative risk analysis, Bayesian modeling, and information theory, to infer the likelihood of catastrophic accidents based upon precursor data.

The application of this methodology is based upon the principle that major accidents and their links to near accidents arise from common initiating events and descriptive factors. As a consequence, the occurrence of such events and descriptive factors conveys essential information about the probability of an extreme accident.

The methodology combines a complex Bayesian model of the events and descriptive factors contributing to a LOS and the application of information theory to quantify the mutual information. This combined methodology allows for an identification of events and descriptive factors with the highest amount of mutual information on near accidents. These events and descriptive factors are later used to establish rough predictive scenarios to anticipate the occurrence of major LOS or MAC. 

### 4.1. Benefits of the Methodology Application

This study illustrates how simple inference methods allow the exploration of information of simple operational errors to predict the likelihood of near accidents. Although there are other sophisticated approaches to the assessment of accident precursors, the added value of this information derives from the fact that near accidents frequently take place prior to major accidents. The method, therefore, allows us to take advantage of an abundance of partially relevant data, which reflect operational issues and errors.

The processes, analyses, and modeling have demonstrated the detection of precursors for serious loss of separation incidents from simple reports and the construction of simple models for future incident prediction and barrier evaluation. 

Within the present case study, a correlation between events and factors is set up and achieves predictive quality, which supports the identity of a set of events and factors that could occur with high probability in a new incident case. 

In summary, the proposed methodology provides an in-depth diagnostic to serious loss of separation scenarios and predictive capacity for new incident analyses.

### 4.2. Limitations of the Application

The application of this methodology is limited, with causes as follows:

- BN model limitation: As well as other predictive models, uncertainties are inevitable in the BN model. The degree of uncertainties can only be reduced if the model is updated with new incident cases constantly; thus, the location of a contrastive event or factor to incident should be more accurate.
- Data source limitation: Even though all events and factors are extracted from the incident reports, the BN is based on expert knowledge due to the quantitative limitation on investigated incident data. Taking into account that all events or factors extracted from incident reports could be occurring in other ATM occurrences not classified as safety incidents, the consequences of this missing real data could affect the accuracy of the Information Theory approach.

### 4.3. Future Work

- Regarding the methodology and case study results, a new reduced ATM safety monitoring program could be designed and implemented for real operations. This real application implies BN model improvement using the real operational data as feedback.
- Radar data can be used to solve the data source limitation problem. Data extracted from FDR and voice communications between pilots and ATCOs contribute to the identification of events or factors present in ATM occurrences without incident categories.

## Figures and Tables

**Table 1:** CPT of events and descriptive factors under scenario of loss of separation in civil/commercial aviation.

Adverse Events (E)	Event Definition	P(E)	Descriptive Factors (DF)	Descriptive Factor Definition	P(DF)	P(DF|E)
1230000	Communication systems related event	5.88 × 10^−2^	12232800	Flight crew’s operation of communication equipment	5.88 × 10^−2^	1.00
2020201	Air navigation service erroneous clearance	1.76 × 10^−1^	22060100	Air traffic management’s monitoring of aircraft	5.88 × 10^−2^	3.33 × 10^−1^
			24010703	Air traffic control provision of flight information	5.88 × 10^−2^	3.33 × 10^−1^
			25050000	Air traffic management service personnel operating procedures/instructions	1.18 × 10^−1^	6.67 × 10^−1^
2020202	Air navigation service clearance to wrong altitude	1.18 × 10^−1^	22120100	Air traffic management’s strategic planning for conflict detection	5.88 × 10^−2^	5.00 × 10^−1^
			24010704	Air traffic control provision of a minimum safe flight level/altitude/height/sector altitude	5.88 × 10^−2^	5.00 × 10^−1^
2020300	Communication between pilot and air navigation service related event	5.29 × 10^−1^	12232800	Flight crew’s operation of communication equipment	5.88 × 10^−2^	1.11 × 10^−1^
			12251800	Flight crew’s radiotelephony phraseology	5.88 × 10^−2^	1.11 × 10^−1^
			12252600	Flight crew’s air/ground/air communication	2.94 × 10^−1^	5.56 × 10^−1^
			22080101	Air traffic management’s internal coordination of civil sectors in the same unit	5.88 × 10^−2^	1.11 × 10^−1^
			24010101	Air traffic control use of phraseology	5.88 × 10^−2^	1.11 × 10^−1^
			24010102	Air traffic control use of readback/hearback error detection	3.53 × 10^−1^	6.67 × 10^−1^
			24010103	Blocked communication	1.18 × 10^−1^	2.22 × 10^−1^
			24010107	Air traffic control requirement for the acknowledgement of information by the flight crew	5.88 × 10^−2^	1.11 × 10^−1^
			24010108	Air traffic control requirement for the acknowledgement of information by the air traffic control officer	5.88 × 10^−2^	1.11 × 10^−1^
			24010301	Frequency selection in the air traffic control communication	5.88 × 10^−2^	1.11 × 10^−1^
			25050000	Air traffic management service personnel operating procedures/instructions	5.88 × 10^−2^	1.11 × 10^−1^
2020400	Controlled/restricted airspace infringement	1.18 × 10^−1^	12251600	Flight crew’s action in respect to air traffic control procedure	5.88 × 10^−2^	5.00 × 10^−1^
			12252400	Flight crew’s action in respect to procedure for transfer to visual flight	5.88 × 10^−2^	5.00 × 10^−1^
			22060100	Air traffic management’s monitoring of aircraft	5.88 × 10^−2^	5.00 × 10^−1^
			24010107	Air traffic control requirement for the acknowledgement of information by the flight crew	5.88 × 10^−2^	5.00 × 10^−1^
2020508	Clearance deviation—approach	1.18 × 10^−1^	12251500	Flight crew’s action in respect to air traffic control clearance	5.88 × 10^−2^	5.00 × 10^−1^
			23020400	Air traffic control use of clearance procedure	5.88 × 10^−2^	5.00 × 10^−1^
			24010107	Air traffic control requirement for the acknowledgement of information by the flight crew	5.88 × 10^−2^	5.00 × 10^−1^
2100200	Diversion due to operational decision	5.88 × 10^−2^	12251500	Flight crew’s action in respect to air traffic control clearance	5.88 × 10^−2^	1.00
4010100	Air navigation services operational communications relate	2.94 × 10^−1^	12252600	Flight crew’s air/ground/air communication	5.88 × 10^−2^	2.00 × 10^−1^
			22080101	Air traffic management’s internal coordination of civil sectors in the same unit	1.18 × 10^−1^	4.00 × 10^−1^
			22080102	Air traffic management’s internal coordination of military sectors in the same unit	5.88 × 10^−2^	2.00 × 10^−1^
			22080202	Air traffic management’s coordination with an adjacent military unit	5.88 × 10^−2^	2.00 × 10^−1^
			24010102	Air traffic control use of readback/hearback error detection	5.88 × 10^−2^	2.00 × 10^−1^
			24010106	Air traffic control transfer of communication	5.88 × 10^−2^	2.00 × 10^−1^
			24010204	Communication technique used by air traffic management in ground to ground communication	5.88 × 10^−2^	2.00 × 10^−1^
4010200	Air navigation services operational information provisions	5.88 × 10^−2^	24010703	Air traffic control provision of flight information	5.88 × 10^−2^	1.00
4010400	Air navigation services conflict detection and resolution related event	5.88 × 10^−1^	22060100	Air traffic management’s monitoring of aircraft	1.76 × 10^−1^	3.00 × 10^−1^
			22080303	Revision of air traffic management’s coordination procedures	5.88 × 10^−2^	1.00 × 10^−1^
			22100600	Briefing for the hand-over/take-over	5.88 × 10^−2^	1.00 × 10^−1^
			22100700	Familiarization with traffic during the hand-over/take-over	5.88 × 10^−2^	1.00 × 10^−1^
			22120100	Air traffic management’s strategic planning for conflict detection	1.76 × 10^−1^	3.00 × 10^−1^
			22120200	Air traffic management’s tactical execution of the conflict detection strategy	2.35 × 10^−1^	4.00 × 10^−1^
			22130101	Air traffic management’s horizontal conflict resolution by radar vectoring/monitoring	5.88 × 10^−2^	1.00 × 10^−1^
			23010300	Clearance procedure	5.88 × 10^−2^	1.00 × 10^−1^
			24010108	Air traffic control requirement for the acknowledgement of information by the air traffic control officer	5.88 × 10^−2^	1.00 × 10^−1^
			24010604	Air traffic control provision of a short-term conflict alert (STCA) warning	5.88 × 10^−2^	1.00 × 10^−1^
			27030000	Air traffic control monitoring of sector traffic load	5.88 × 10^−2^	1.00 × 10^−1^
4010600	Air navigation services handing over/taking over procedure	1.18 × 10^−1^	22080101	Air traffic management’s internal coordination of civil sectors in the same unit	1.18 × 10^−1^	1.00
			23010300	Clearance procedure	5.88 × 10^−2^	5.00 × 10^−1^
4050400	Failure of data processing	5.88 × 10^−2^	22060100	Air traffic management’s monitoring of aircraft	5.88 × 10^−2^	1.00
			24010705	Air traffic control provision of delay related information	5.88 × 10^−2^	1.00
4070400	Air space capacity reduction	2.94 × 10^−1^	12251500	Flight crew’s action in respect to air traffic control clearance	5.88 × 10^−2^	2.00 × 10^−1^
			22080103	Air traffic management’s internal coordination of positions in civil sectors in the same unit	5.88 × 10^−2^	2.00 × 10^−1^
			22100300	Airspace during the hand-over/take-over	5.88 × 10^−2^	2.00 × 10^−1^
			24010107	Air traffic control requirement for the acknowledgement of information by the flight crew	5.88 × 10^−2^	2.00 × 10^−1^
			27030000	Air traffic control monitoring of sector traffic load	1.18 × 10^−1^	4.00 × 10^−1^
			27050200	Factors relating coordination with air traffic flow management	5.88 × 10^−2^	2.00 × 10^−1^
			41100300	Runway obstruction	5.88 × 10^−2^	2.00 × 10^−1^
			52020400	Tailwind	5.88 × 10^−2^	2.00 × 10^−1^
			52031400	Cloud amount restricting visibility	5.88 × 10^−2^	2.00 × 10^−1^

**Table 2:** Severity A incidents occurred in last year.

Adverse Events (E)	Event Definition	Descriptive Factors (DF)	Descriptive Factor Definition
2020300	Communication between pilot and air navigation service related event	12252600	Flight crew’s air/ground/air communication
		22080101	Air traffic management’s internal coordination of civil sectors in the same unit
		24010102	Air traffic control use of readback/hearback error detection
4010400	Air navigation services conflict detection and resolution related event	22120100	Air traffic management’s strategic planning for conflict detection
		22120200	Air traffic management’s tactical execution of the conflict detection strategy
		23010300	Clearance procedure
4010600	Air navigation services handing over/taking over procedure	22080101	Air traffic management’s internal coordination of civil sectors in the same unit

**Table 3:** Evaluation of predictive scenarios.

Scenario	DFs in Each Scenario	TPR	FPR	ACC
1	24010102: Air traffic control use of readback/hearback error detection	0.420	0.420	0.580
2	22120200: Air traffic management’s tactical execution of the conflict detection strategy	0.320	0.320	0.680
3	22080101: Air traffic management’s internal coordination of civil sectors in the same unit	0.370	0.370	0.630
4	12252600: Flight crew’s air/ground/air communication	0.370	0.370	0.630
5	22120100: Air traffic management’s strategic planning for conflict detection	0.320	0.320	0.680
6	22060100: Air traffic management’s monitoring of aircraft	0.320	0.320	0.680
7	24010102+22060100: Air traffic control use of readback/hearback error detection & Air traffic management’s monitoring of aircraft	0.740	0.155	0.845
8	24010102+22060100+22080101: Air traffic control use of readback/hearback error detection & Air traffic management’s monitoring of aircraft & Air traffic management’s internal coordination of civil sectors in the same unit	0.790	0.050	0.950
9	24010102+22060100+22120100: Air traffic control use of readback/hearback error detection & Air traffic management’s monitoring of aircraft & Air traffic management’s strategic planning for conflict detection	0.840	0.043	0.957
10	24010102+22120200+22080101+12252600+22120100+22060100: All six precursors	0.950	0.002	0.998

## Abbreviations

A/C	Aircraft
ACC	Accuracy
ADREP	Accident/Incident Data Reporting
ATCO	Air Traffic Control Officer
ATM	Air Traffic Management
ATS	Air Traffic Services
BN	Bayesian Network
CPT	Conditional Probabilistic Table
DAG	Directed Acyclic Graph
DF	Descriptive Factor
EASA	European Aviation Safety Agency
EF	Explanatory Factor
EU	European Union
FDM	Flight Data Monitoring
FDR	Flight Data Recorder
FN	False Negatives
FP	False Positives
FPR	False Positive Rate
ICAO	International Civil Aviation Organization
LOO	Leave One Out
LOS	Loss of Separation
MAC	Mid-Air Collisions
NextGen	Next Generation Air Transportation System
ROC	Receiver Operating Characteristic
SESAR	Single European Sky ATM Research
SOAM	Safety Occurrence Analysis Methodology
STCA	Short Term Conflict Alert
TN	True Negatives
TP	True Positives
TPR	True Positive Rate
